# Retrospective analysis: The application of human menopausal gonadotropin combined with letrozole for IUI in patients undergoing artificial insemination by husband due to unexplained or mild male factors

**DOI:** 10.3389/fendo.2022.1038433

**Published:** 2022-12-20

**Authors:** Hua-qing Li, Xin-ling Pan, Nian-jun Su, Xi-ping Lu, Jun-qing Chen, Xu-wei Chen

**Affiliations:** ^1^ Department of Reproductive Center, Affiliated Dongyang Hospital of Wenzhou Medical University, Dongyang, Zhejiang, China; ^2^ Department of Biomedical Sciences Laboratory, Affiliated Dongyang Hospital of Wenzhou Medical University, Dongyang, Zhejiang, China; ^3^ Department of Reproductive Health and Infertility, Guangdong Women and Children Hospital, Guangzhou, China; ^4^ Department of Gynecology, Affiliated Dongyang Hospital of Wenzhou Medical University, Dongyang, Zhejiang, China

**Keywords:** letrozole, human menopausal gonadotropin, ovarian stimulation, intrauterine insemination, artificial insemination by husband

## Abstract

**Objective:**

To compare the effects of human menopausal gonadotropin (HMG) combined with letrozole (LE) to HMG only for ovarian stimulation on pregnancy outcome of infertile patients undergoing artificial insemination by husband (AIH) due to unexplained or mild male factors.

**Materials and methods:**

Infertile patients with unexplained or mild male factors treated from July 2015 to December 2021 were selected as subjects. The patients were divided into two groups according to the ovarian stimulation schemes they received, namely HMG combined with LE or HMG only. We analyzed the laboratory examination results before drug treatment (baseline) and during ovarian stimulation and compared the pregnancy outcomes of the two groups using univariable analysis and multivariable logistic regression analysis.

**Results:**

In total, 526 cycles of 372 couples were included. The univariate analysis showed that the clinical pregnancy rate of the HMG combined with LE group was 24.8%, significantly higher than that of the HMG group (14.8%, P = 0.007). The live birth rate (19.9%) of the HMG combined with LE group were also significantly higher than those of the HMG group (11.2%, respectively). In multivariate logistic analysis, the age of males was negatively associated with the clinical pregnancy rate (OR 0.874, 95% CI 0.793~0.963, P=0.006) and live birth (OR0.875, 95% CI 0.783~0.977, P=0.018). Moreover, ovarian stimulation with HMG+LE was the only beneficial factor significantly associated with clinical pregnancy (OR 1.929, 95% CI 1.068~3.485, P=0.029) and live birth (OR 2.255, 95% CI 1.188~4.282, P=0.013).

**Conclusion:**

Ovarian stimulation using HMG combined with LE can increase the clinical outcomes (live birth and clinical pregnancy) among infertile patients undergoing AIH due to explained or mild male factors.

## 1 Introduction

Infertility is defined as the inability of a couple maintaining a regular sexual life without contraception to conceive within one year (1). Intrauterine insemination (IUI) is one of the most widespread types of assisted conception. It increases the probability of conception in infertile couples. This technique is simple, fast, low-cost and manageable, with a relatively low incidence of complications (2). In many countries, IUI is recommended as the first-line treatment for infertility, including those cases with unexplained or mild male factor (2, 3).

In the process of IUI, ovarian stimulation (OS) is routinely performed in couples with infertility due to unexplained and mild male factors (4–6). Either the single use of letrozole (LE) or the single use of human menopausal gonadotropin (HMG) can be used to induce ovulation. LE is a third-generation aromatase inhibitor, which usually causes mono-ovulation. HMG consists of the follicle stimulating and luteinizing hormones, which play a key role in the development of follicles. However, both drugs have certain limitations. Ovarian stimulation by HMG alone may cause multi-follicular development, leading to excessive estrogen levels and promoting endometrial hyperplasia. In turn, these can result in several complications such as multiple pregnancy, ovarian hyperstimulation syndrome, and endometrial thickening. Furthermore, HMG is usually administered as daily injections in a hospital environment, making it inconvenient and relatively painful. Although LE is an oral drug and thus more advantageous, its efficiency duration does not last long because of LE’s short half-life duration. This phenomenon causes the dosage of LE alone not to be sufficient to promote the development of dominant follicles. Therefore, ovulation stimulation needs to be repeated during the next menstrual cycle with a higher LE dosage.

Due to these issues, finding an OS protocol that can improve the clinical pregnancy rate while also reducing the multiple pregnancy rate has become a goal of many physicians. In recent years, single use of LE has been recommended for anovulatory women and has achieved a high successful rate, thus it is widely used in controlled ovarian stimulation (7). Hence, we propose that HMG combined with LE (HMG + LE) can reduce the multiple pregnancy rate without affecting the clinical pregnancy rate and live birth rate. To explore this hypothesis, a retrospective study was conducted in a tertiary hospital in Southeast China.

## 2 Materials and methods

### 2.1 Inclusion and exclusion criteria of subjects

**Figure 1:**
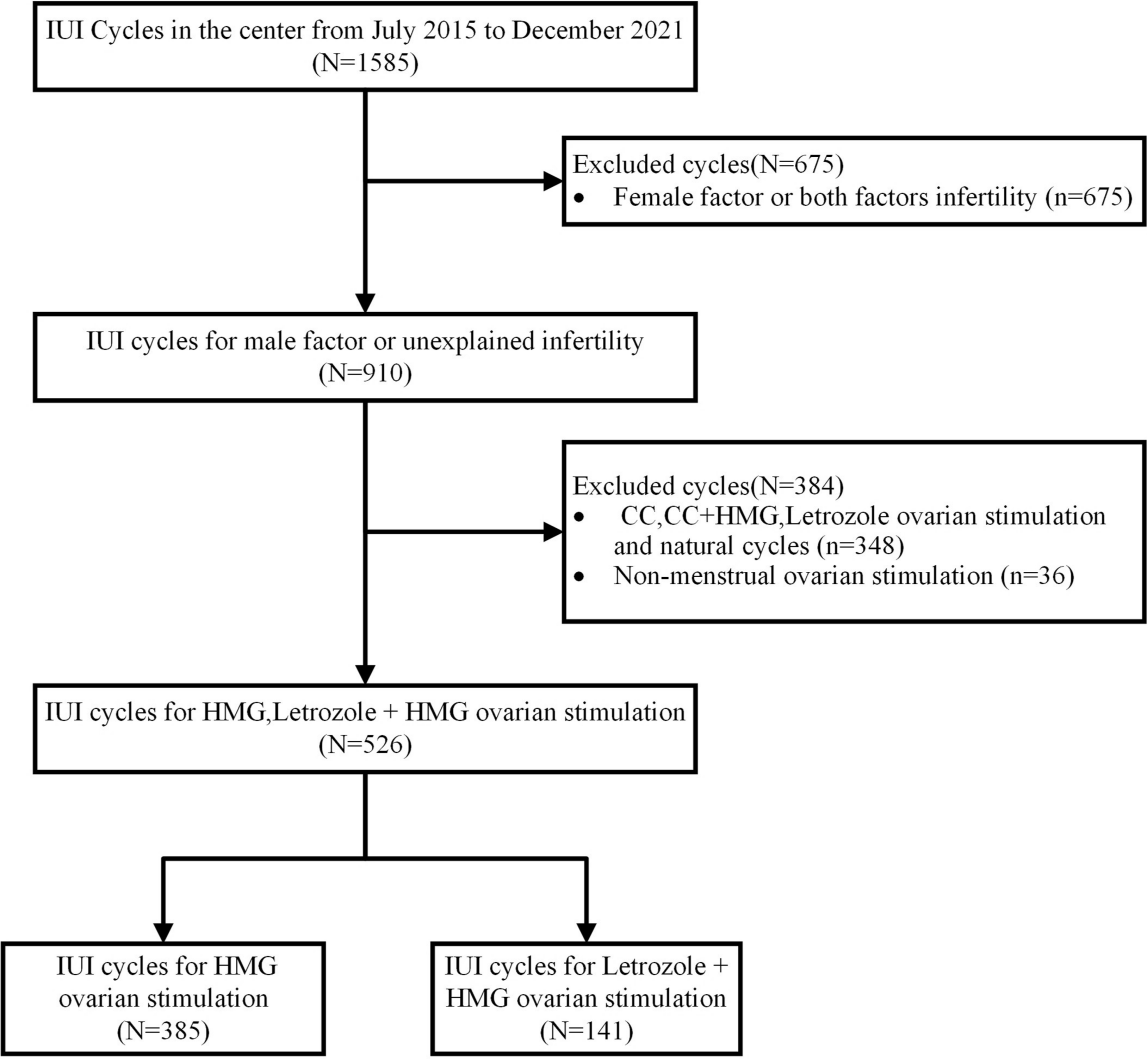
Inclusions of identifiable human data.

Patients undergoing artificial insemination by husband (AIH) in our hospital from July 2015 to December 2021 were retrospectively collected (**Figure 1**). These couples received AIH because they were unable conceive after one year or more of regular sexual intercourse without contraception. After excluding cases of abnormal ovulation, endocrine diseases, intrauterine adhesions, and uterine malformations, their conditions were confirmed as unexplained infertility or mild male factor. Informed consent was obtained from the involved patients prior to the study. This study was approved by the Ethics Committee and Institutional Review Board of Dongyang People’s Hospital.

### 2.2 Intervention of research objects

#### 2.2.1 OS group

Female patients started to use HMG (trade name: Lebaode, Livzon Group Livzon Pharmaceutical Co, Ltd., Zhuhai, China) + LE (trade name: Fu Rui, Hengrui Medicine Co, Ltd., Jiangsu, China) or HMG only to perform OS. The OS regimen was conducted according to the guidelines of ovarian stimulation in assisted reproductive technology in China. Briefly, patients in the HMG only group were administered with HMG on the 2^nd^-6^th^ day of the menstruation circle (early follicular phase). The initial dose was 75 mg per day but was increased to 112.5 mg if there was no development in follicular diameters and hormones after 5-7 days since the initial stimulation. Patients in the HMG+LE group were administered with 2.5-5.0 mg of LE per day on the 2^nd^ -6^th^ day of the menstruation cycle and the duration would last for 3-5 days. HMG was then administered in the same dosage as in the HMG only group if the follicle did not mature until the mature follicle formed.

#### 2.2.2 Follicle monitoring and laboratory examination

The follicular diameters and hormones, including follicle stimulating hormone, luteinizing hormone, estradiol, and progesterone, were detected on the third day of menstruation as baseline data. In all treatment cycles, follicles were continuously monitored from the 9th day of the menstrual cycle. When the follicle diameter reached ≥ 14 mm, the levels of luteinizing hormone, estradiol and progesterone were monitored. After the peak of estradiol appeared or luteinizing hormone surged, 10000 units of human chorionic gonadotropin (HCG) or 0.2 mg of triptorelin acetate were injected for triggering, and 12h and 36h later, AIH was conducted.

#### 2.2.3 Semen processing and AIH performance

After sexual abstinence for 3–7 days, the husband collected semen through masturbation in a collection cup. The semen parameter (total progressive motile sperm count (TPMSC) was recorded. The semen was processed using density gradient centrifugation followed by AIH in the uterine cavity.

#### 2.2.4 Corpus luteum support

After ovulation, progesterone (200 mg/D) or dydrogesterone (20 mg/D) were administrated continuously for 14 days.

### 2.3 Determination of pregnancy outcome

Urine HCG (positive) or blood HCG (> 10 mIU/mL) was examined 14 days after IUI to determine whether the patients were biochemically pregnant. A clinical pregnancy was defined as the presence of a gestational sac upon transvaginal ultrasonography after three to four weeks following AIH. An ectopic pregnancy was diagnosed by either laparoscopy or sonographic visualization of an extrauterine gestational sac (8). Early miscarriage was defined as pregnancy loss that occurred spontaneously before 12 weeks of gestation (9). When ultrasonography findings showed two or more gestational sacs in the uterus, a multiple pregnancy was suspected. Live birth was defined as the delivery of a living fetus (or living fetuses) beyond 28 weeks of gestation (10).

### 2.4 Statistical analysis

SPSS 26.0 (IBM Corporation, USA) was used for statistical analysis of the data. The data conforming to normal distribution in continuous variables were expressed as mean ± SD; otherwise, they were expressed as median and quartile. The differences between groups were analyzed using univariate analysis; *t*-test for continuous variables with normal distribution, Mann-Whitney U test for continuous variables with abnormal distribution, and chi square test for categorical variables. The significant variables in univariate analysis were included in a multivariate binary logistic regression analysis (ENTER method). The results were expressed as odds ratio (OR) with a 95% confidence interval (CI). A P value < 0.05 was considered statistically significant.

## 3 Results

### 3.1 Comparison of patient characteristics between the LE+HMG and HMG group

A total of 372 couples and 526 cycles were included in this study. The average age of the females was 30 years old, with an average duration of infertility of two years. Among all the cycles, 236 cycles corresponded to primary infertility and 290 cycles to secondary infertility. There were 141 cycles in the HMG+LE group and 385 cycles in the HMG group. The basic characteristics of the patients are shown in **Table 1**. We observed that the ages of females and males were significantly different between the two groups (P<0.05). There were lower baseline levels of FSH and TPMSC and a higher level of LH in HMG+LE group compared to the HMG group. In addition, the percentage of dydrogesterone for luteal support in HMG+LE group was 84.4%, which was significantly higher than that of the HMG group (59.2%; P<0.001). There was no significant difference between the two groups in trigger drugs, female body weight, female body mass index, type of infertility, and duration of infertility (P>0.05).

**Table 1:** The basic characteristics of the patients receiving human menopausal gonadotropin (HMG) with letrozole and HMG alone.

Variables	Total	HMG	HMG+LE	p
	(n = 526)	(n = 385)	(n = 141)	
Female age (year)	30.0 (27.0, 33.0)	30.0 (27.0, 34.0)	29.0 (27.0, 32.0)	0.005
Male age (year)	32.0 (29.0, 36.0)	32.0 (29.0, 36.0)	31.0 (28, 35.0)	0.015
Type of Infertility, n (%)				0.084
Primary	236(44.9)	164 (42.6)	72 (51.1)	
Secondary	290(55.1)	221 (57.4)	69 (48.9)	
Duration of infertility (year)	2.0 (1.0, 3.0)	2.0 (1.0, 3.0)	2.0 (2.0, 4.0)	0.254
Female weight (kg)	55.0 (50.0, 61.0)	55.0 (50.0, 60.0)	56.0 (50.0, 60.0)	0.13
Female body mass index (kg/m2)	21.7 (19.8, 24.1)	21.5 (19.7, 24.0)	22.0 (19.9, 25.0)	0.095
Baseline sex hormone				
Follicle stimulating hormone (IU/L)	6.3 (5.5, 7.4)	6.6 (5.6, 7.5)	5.9 (5.2, 6.7)	< 0.001
Luteinizing hormone (IU/L)	4.9 (3.8, 6.7)	4.8 (3.7, 6.3)	5.9 (4.4, 8.4)	< 0.001
Estradiol (pmol/L)	114.0 (90.0, 160.7)	115.8 (91.4, 157.9)	113.3 (89.0, 164.1)	0.787
Progesterone (nmol/L)	1.2 (0.8, 1.8)	1.3 (0.8, 1.8)	1.0 (0.8, 1.8)	0.146
TPMSC (million)	45.8 (24.4, 72.0)	47.2 (25.7, 74.8)	39.6 (22.4,63)	0.032
Luteal support, n (%)				< 0.001
Progesterone	179 (34.0)	157 (40.8)	22 (15.6)	
Dydrogesterone	347 (66.0)	228 (59.2)	119 (84.4)	
Trigger drug, n (%)				0.086
HCG	464 (88.9)	335 (87.5)	129 (92.8)	
Triptorelin acetate	58 (11.1)	48 (12.5)	10 (7.2)	

TPMSC, total progressive motile sperm count.

### 3.2 Hormonal parameters after OS between the LE+HMG and HMG group

As shown in **Table 2**, the number of dominant follicles in the HMG group was significantly higher than that in HMG+LE group. In terms of the HMG group, the medication duration, dosage of HMG, and endometrial thickness on the day of triggering were higher than those in HMG + LE group, with statistical significance (P<0.001). Estradiol in the HMG group (1738.0 _pmol/L) on the day of triggering was higher than that in HMG + LE group (942.7pmol/L), with statistical significance (P<0.001). The luteinizing hormone on the day of triggering in the HMG group (11.2 IU/L) was lower than that in HMG + LE group (16.2 IU/L), with statistical significance (P<0.001). Notably, there was no significant difference in progesterone between the two groups on the day of triggering (P = 0.978).

**Table 2:** The impact of different ovarian stimulation regimen on the status before IUI.

Variables	HMG (n = 385)	HMG+ LE (n = 141)	P
Number of dominant follicles	1.0 (1.0, 2.0)	1.0 (1.0, 2.0)	< 0.001
Dose of HMG^a^ (U)	750.0 (525.0, 975.0)	375.0 (225.0, 525.0)	< 0.001
Duration of HMG^b^ (day)	9.0 (7.0, 10.0)	5.0 (3.0, 7.0)	< 0.001
Endometrial thickness^c^ (mm)	10.0 (8.6, 11.8)	9.0 (8.0, 10.4)	< 0.001
Luteinizing hormone^c^ (IU/L)	11.2 (6.5, 19.2)	16.2 (9.0, 32.7)	< 0.001
Estradiol^c^ (pmol/L)	1738.0 (1126.0, 3116.0)	942.7 (636.9, 1408.0)	< 0.001
Progesterone^c^ (nmol/L)	1.6 (1.1, 2.5)	1.6 (1.2, 2.2)	0.978

^a^the accumulative amount of HMG before triggering.

^b^the duration of HMG administration.

^c^the concentration on the day of triggering.

### 3.3 The pregnancy outcomes between the LE+HMG and HMG group

The clinical pregnancy rate and live birth rate of the HMG + LE group were 24.8% and 19.9%, significantly higher than those in the HMG group (14.8% and 11.2%, respectively). However, there were no significant differences in the biochemical pregnancy rate, multiple pregnancy rate, early miscarriage rate, ectopic pregnancy rate, and singleton pregnancy rate between the two groups (**Table 3**). Notably, there was no case with ovarian hyperstimulation syndrome in this study.

**Table 3:** Comparison of pregnancy outcomes between the HMG + LE group and the HMG only group.

Variables	HMG n (%)	HMG +LE n (%)	P
Clinical pregnancy	57 (14.8)	35 (24.8)	0.019
Biochemical pregnancy	6 (1.6)	3 (2.1)	0.672
Early miscarriage	14 (3.6)	4 (2.8)	0.542
Ectopic pregnancy	2 (0.5)	3 (2.1)	0.190
Single pregnancy	49 (12.7)	27 (19.1)	0.092
Multiple pregnancies	6 (1.6)	6 (4.3)	0.371
Live birth	43 (11.2)	28 (19.9)	0.002

### 3.4 The factors associated with clinical pregnancy and live birth

Triggering drug was included in multivariate analysis despite the factor not being significant in the univariate analysis because of its importance. Of note, the age of males was negatively significantly associated with clinical pregnancy (OR 0.874, 95% CI 0.793~0.963, P=0.006) and live birth (OR0.875, 95% CI 0.783~0.977, P=0.018) (**Table 4**). LE+HMG usage increased the clinical pregnancy (OR 1.929, 95% CI 1.068~3.485, P=0.029) and live births (OR 2.255, 95% CI 1.188~4.282, P=0.013). The baseline hormone levels, including FSH and LH, drugs for luteal support and triggering, and semen parameter (TPMSC), were not significantly associated with clinical outcomes (live birth and clinical pregnancy).

**Table 4:** Analyses of factors affecting clinical pregnancy rate and live birth rate using multi-variable logistic regression model.

Variable	clinical pregnancy		live birth	
	OR	P	OR	P
	(95% CI)		(95% CI)	
Female age (year)	1.102 (0.997~1.217)	0.056	1.087 (0.970~1.217)	0.150
Male age (year)	0.874 (0.793~0.963)	0.006	0.875 (0.783~0.977)	0.018
OS regimen n (%)				
HMG	1		1	
HMG+LE	1.929(1.068~3.485)	0.029	2.255 (1.188~4.282)	0.013
Follicle stimulating hormone^a^ (IU/mL)	1.018 (0.871~1.189)	0.827	1.063 (0.894~1.264)	0.487
Luteinizing hormone (IU/L)	0.988 (0.935~1.044)	0.672	0.985 (0.927~1.047)	0.638
Luteal support regimen n (%)				
progesterone	1		1	
dydrogesterone	1.359 (0.784~2.356)	0.274	1.845 (0.967~3.520)	0.063
TPMSC (million)	0.999 (0.994~1.004)	0.690	0.999 (0.994~1.005)	0.849
Trigger drug, n (%)				
	1		1	
	1.066 (0.467~2.430)	0.880	1.105 (0.434~2.816)	0.834

^a^Baseline sex hormone.

## 4 Discussion

The effects of HMG or LE alone in artificial insemination have been confirmed in previous studies. However, whether the combination of both would get more benefit remains unknown. In this retrospective observational study, the HMG+LE and HMG OS regimens were adopted to improve the clinical outcomes of infertile patients whose infertility was attributed unexplained or mild male factors. Patients stimulated with HMG+LE had better clinical outcomes than those stimulated with HMG only after adjusting differences in baseline characteristics, semen parameter, trigger drugs, and luteal support.

OS in infertile patients can increase the success probability of artificial insemination and clinical pregnancy rate. The commonly used drugs for OS include clomiphene, LE, HMG, urinary FSH, and GnRH agonists (11–15). A recent meta-analysis compared the effectiveness of clomiphene, LE, HMG, and different natural cycle regimens in the treatment of unexplained infertility (16). The results revealed that OS using HMG ranked the highest in terms of the live births/ongoing pregnancy rate, while natural cycle ranked the lowest. HMG has thus been commonly used for OS by patients receiving IUI (17, 18). Letrozole is a third-generation aromatase inhibitor and is combined with HMG, resulting in effective ovary stimulation with lower cost because it lowers the dosage of HMG (19, 20). There are several mechanisms explaining how LE increases ovary sensitivity to HMG. Letrozole inhibits the conversion of androgen to estrogen in the peripheral ovarian tissues by inhibiting aromatase activity, resulting in a transient accumulation of androgen. Consequently, the increased androgen improves the expression of insulin-like growth factor 1, which increases the response sensitivity of the ovaries to HMG (19). Moreover, low estrogen levels may reduce the ubiquitination of estrogen receptors and lead to faster endometrial proliferation and increased blood flow to the uterus and endometrium, which also has a positive impact on pregnancy outcomes (21). This study found that patients stimulated with HMG+LE had a higher live birth rate than those in the HMG group, confirming the better outcome of HMG + LE as we hypothesized. The higher birth rate in the combination regimen is attributed to an improvement in endometrium condition (22), and should be adopted in patients receiving frozen embryo for endometrial preparation (23). Though clomiphene also has effects on endometrium, its usage in ovary stimulation is associated with lower clinical pregnancy rates than HMG+LE (24).

Previous studies postulate that HMG alone causes significantly higher rates of multiple pregnancy compared to other drug regimens (16). Notably, this issue can potentially be solved by combining it with other ovarian stimulation drugs. Letrozole does not consume estrogen receptors in the brain and retains the reserved normal negative feedback function to control the elevated estrogen levels and growth of dominant follicles (25, 26). Only a single follicle is thus expelled in most cases. The rate of multiple pregnancy can thus be theoretically reduced (22, 23) when HMG is combined with letrozole. However, there was no statistical significance in the rate of multiple pregnancy in this study. This phenomenon was attributed to the limited sample size of multiple pregnancy cases in this retrospective study.

The clinical outcomes following IUI are influenced by many factors, including age, infertility type, sperm quality, mature follicular number, and endometrial thickness among other factors (24–28). Herein, the age of males was negatively associated with the clinical pregnancy and live birth in this study, indicating that it also plays a key role in clinical outcomes in patients with infertility attributed to unexplained or mild male factors (29). Moreover, different ovulatory drugs were found to be the only factor affecting the live birth rate after adjusting the confounding factors through multivariate binary regression analysis, highlighting the main role of ovarian stimulation in determining the final clinical outcome. The clinical outcomes in patients receiving IUI treatment are determined by several parameters, including clinical pregnancy, biochemical pregnancy, and live birth. Live birth might be adopted as the finical outcome because the other two might suffer from miscarriages. In this study, the live birth rate was only associated with ovarian stimulation, with patients receiving HMG combined with LE exhibiting better results compared to those receiving HMG only.

The limitation of the study is that it is a retrospective study conducted in a single center and thus possible bias in determination of different ovarian stimulation by the doctors may exist.

## 5 Conclusion

HMG+LE is superior to HMG in patients with infertility attributed to unexplained or mild male factors because it induces higher clinical pregnancy and live birth rates. However, the age of males was the negative factor for clinical outcomes. HMG + LE should thus be used for ovarian stimulation in patients receiving intrauterine insemination.

## Data availability statement

The original contributions presented in the study are included in the article/supplementary material. Further inquiries can be directed to the corresponding author.

## Ethics statement

The studies involving human participants were reviewed and approved by Ethics Committee and Institutional Review Board of Dongyang People’s Hospital. Written informed consent for participation was not required for this study in accordance with the national legislation and the institutional requirements.

## Author contributions

(I) Conception and design: H-QL, N-JS, J-QC; (II) Administrative support: J-QC, X-PL; (III) Provision of study materials or patients: X-PL, H-QL; (IV) Collection and assembly of data: H-QL, X-LP, X-WC; (V) Data analysis and interpretation: H-QL, X-LP; (VI) Manuscript writing: J-QC, H-QL; (VII). All authors contributed to the article and approved the submitted version.
